# Medial Pelvic Migration of the Lag Screw after Intramedullary Nailing for Trochanteric Femoral Fracture

**DOI:** 10.1155/2021/5553835

**Published:** 2021-05-28

**Authors:** Kohei Kuroshima, Koichi Kasahara, Shinsuke Kihara, Yoshifumi Harada, Masatoshi Sumi

**Affiliations:** ^1^Department of Orthopaedic Surgery, Kobe Rosai Hospital, 4-1-23 Kagoike-dori, Chuo-ku, Kobe, Hyogo 651-0053, Japan; ^2^Department of Orthopaedic Surgery, Konan Medical Center, 1-5-16 Kamokogahara, Higashinada-ku, Kobe, Hyogo 658-0064, Japan

## Abstract

Internal fixation with intramedullary nails has gained popularity for the treatment of trochanteric femoral fractures, which are common injuries in older individuals. The most common complications are lag screws cut-out from the femoral head and femoral fracture at the distal tip of the nail. Herein, we report a rare complication of postoperative medial pelvic migration of the lag screw with no trauma. The patient was subsequently treated by lag screw removal via laparoscopy. This case suggests that optimal fracture reduction, adequate position of the lag screw, and careful attention to set screw insertion are important to prevent complications. Additionally, laparoscopic surgery might be able to remove the lag screw more safely than removal from the femoral side.

## 1. Introduction

Trochanteric femoral fractures are common injuries in older individuals. In 2000, there was an estimated global incidence of nine million osteoporotic fractures, of which 1.6 million were hip fractures [1]. Approximately half of reported hip fractures are extracapsular, and fixation with a dynamic hip screw or intramedullary fixation has become the most favorable treatment [2]. Treatment with a gamma nail is therefore a frequently used method in trauma surgery; however, certain complications, such as lag screws cut-out from the femoral head and femoral fracture at the distal tip of the nail, have been reported [3]. Thus, we present a rare complication of postoperative lag screw migration into the pelvis with no trauma. The patient was subsequently treated with implant removal.

## 2. Case Presentation

**Figure 1:**
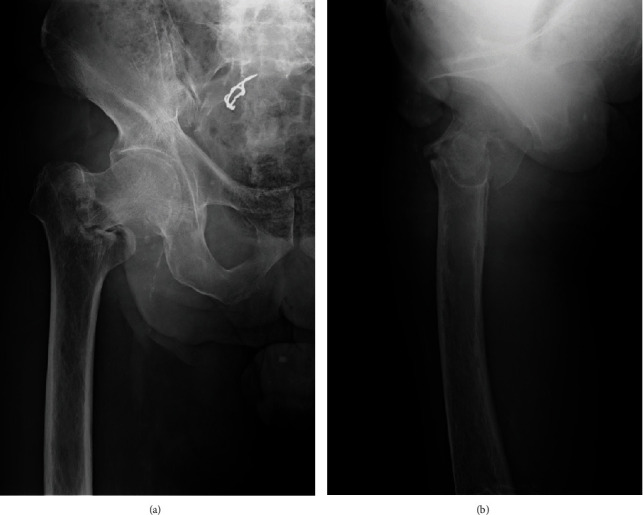
Anteroposterior (a) and lateral (b) radiographs taken on initial presentation showing a trochanteric fracture with a greater trochanteric fragment in the right femur, which are classified as AO type 31A2.2 and Jensen type 3.

**Figure 2:**
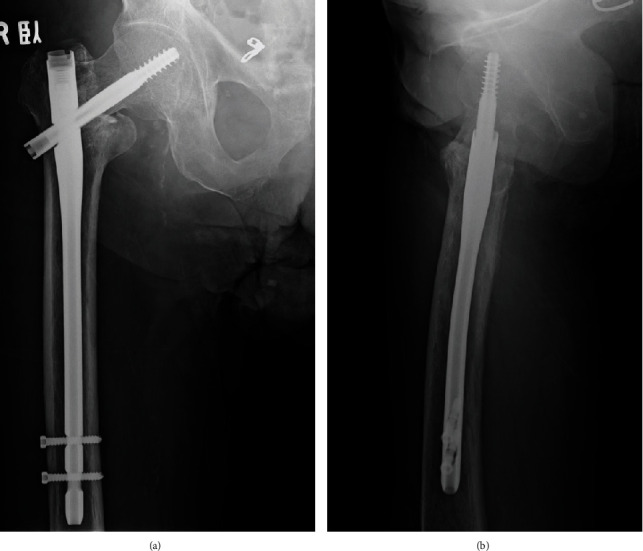
Radiographs after the osteosynthesis of the right trochanteric fracture. Anteroposterior (a) and lateral (b) views of the right femur showing insufficient reduction and poor placement of the lag screw after implantation of a gamma 3 long nail.

**Figure 3:**
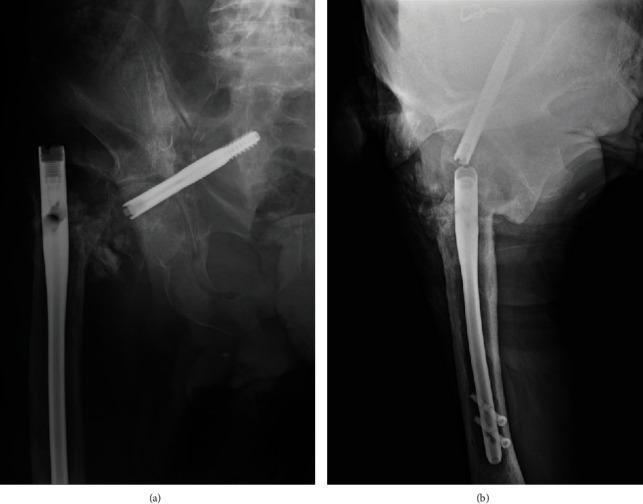
Radiographs showing redisplacement of the fracture and intrapelvic migration of the lag screw through the femoral head and the medial wall of the acetabulum.

**Figure 4:**
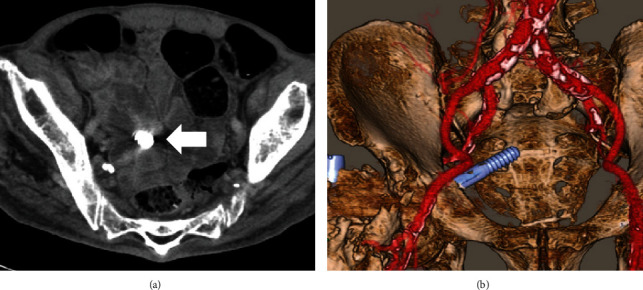
A contrast-enhanced computed tomography scan showing the lag screw located deep in the pelvis, between the internal and external iliac vessels, and tangent to the intestine. (a) Axial view (arrow, lag screw) and (b) reconstructed 3D image.

An 82-year-old man with a history of schizophrenia fell and was transferred to our hospital by ambulance. Upon initial physical examination, tenderness and swelling were noted over the right trochanteric region. His right leg was immobile because of severe right hip pain. Moreover, he had no neurovascular deficits or other extremity or systemic injuries. Radiography revealed an AO type 31A2.2 [4] and Jensen type 3 [5] trochanteric fracture in the right femur (Figure 1). For treatment, the patient underwent a closed reduction and internal fixation with a gamma 3 long nail (Stryker, Kalamazoo, Michigan, US) according to the manufacturer's instructions. The lag screw was the appropriate length and reached to the subchondral bone of the femoral head. However, the lag screw was inserted anteriorly and a varus malalignment remained (Evans classification was type 1 group 3) in AP view (Figure 2) [6]. Complications did not occur during the surgery or throughout the 1-month period of hospitalization. The patient was an elderly and had a history of schizophrenia; hence, rehabilitation did not proceed after surgery. Thus, he was unable to walk by himself and was wheelchair-bound when he was discharged. One year after surgery, the patient presented to the clinic for a regular follow-up. Although there were no additional falls or subjective symptoms, radiograph revealed that the intramedullary nail was disassembled with bone fragment displacement. The lag screw penetrated into the pelvis through the femoral head and acetabulum (Figure 3). No intra-abdominal organ injuries were confirmed on a contrast-enhanced computed tomography scan. The lag screw was located between the internal and external iliac vessels, and tangent to the small intestine; however, no signs of pneumoperitoneum or hematuria were present (Figure 4).

**Figure 5:**
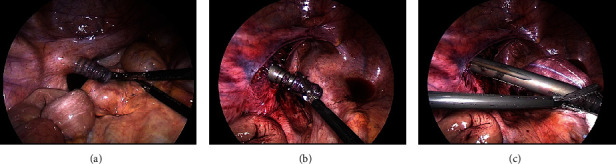
Laparoscopic views showing the lag screw, intestine, and peritoneum: (a) damage to the intestine was not observed; (b) the peritoneum around the lag screw was peeled; (c) the lag screw was pulled out with forceps.

To prevent future concomitant injury of the blood vessels, ureter, and intestine, we decided to remove the implants. If the lag screw had been adhering to the intestine, the threaded part of the screw could damage the intestine when removing the lag screw from the femoral side. Therefore, we considered that removing the lag screw under direct view from the pelvic side would be safer. Laparoscopic removal of the lag screw was then performed by gastrointestinal surgeons. Intestinal damage was not observed, and after peeling the peritoneum around the lag screw, the screw was pulled out with forceps (Figure 5). The nail and distal locking screw were then removed by orthopedic surgeons. Furthermore, it was found that the set screw was not inserted to the proper depth. Considering invasion and the fact that he was already unable to walk, we decided to perform implant removal rather than hip replacement.

**Figure 6:**
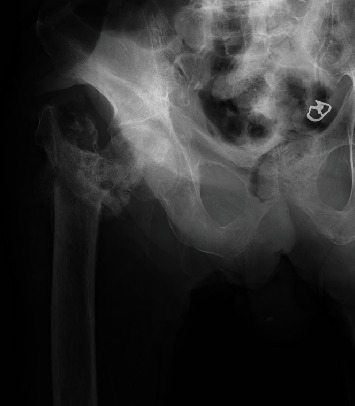
A radiograph at 1 year after implant removal showing nonunion of the right trochanter of the femur.

The postoperative period was uneventful. At 1 year postoperatively, the patient remained wheelchair-bound without pain. A follow-up radiograph revealed nonunion of the right trochanter of the femur (Figure 6).

## 3. Discussion

Commonly accepted operative predictors for fixation failure include the quality of reduction, tip apex distance (TAD), and lag screw position within the femoral head and neck [7]. Rebuzzi et al. reported that out of 981 intramedullary hip fracture fixations, 21 had a “risky” screw position; among those cases, 9 lag screws had cut out and only 3 had penetrated the acetabulum. In general, medial migration is a rare complication (<1%); however, the complication rate can increase up to 43% when the screw is placed in a risky area [8].

Flint et al. classified the risk factors for femoral head and acetabulum penetration and medial migration of lag screws into five categories: (i) intraoperative surgeon-related factors (damage of the femoral head by overreaming or malposition of lag screw), (ii) intraoperative fracture-related factors (lateral buttress deficiency, unstable medial cortex, malreduction in various positions, osteoporotic bone, or distraction of fracture site due to nonweight bearing restrictions), (iii) implant-related factors (nail/lag screw dysfunction, set screw dysfunction, or nail toggling), (iv) technical errors (significant TAD, superior/anterior placement of lag screw in the femoral head, or inadequate lag screw length), and (v) postoperative factors (fall, additional trauma, or abnormal forces to hip articulation) [9]. In our case, the lag screw was fixed on the anterior area and a varus malalignment remained in AP view. Thus, insufficient reduction and poor placement of the lag screw were considered to be potential causes of the medial pelvic migration. In terms of the mechanism underlying the medial migration, Weil et al. demonstrated that repeated axial loading during unstable fixation caused toggling of the nail within the femoral canal and led to medial migration of the lag screw [10]. Moreover, Thein et al. reported that in cases of a greater trochanteric fragment, the nail was often inserted from the fracture part, and toggling of the nail was likely to occur because the bone support structure of the proximal nail was lost [11]. In our case, we considered using a long gamma 3 nail instead of a short nail in order to mitigate the risk of nail toggling, which was high because of a greater trochanteric fragment and a stovepipe canal. Of note, Yoshida and Matsuya suggested that a nail with a large diameter or a long nail was effective at preventing nail toggling because longer nails are able to reduce the intramedullary space for movement [12]. However, despite using a long nail, the migration of the lag screw did occur in our case. This consequence implied that even when long nails are used, minor toggling cannot be prevented in the case of a greater trochanteric fragment or a stovepipe canal. If we had used a nail with a larger diameter, as suggested by Yoshida et al., we might have prevented the nail toggling.

The gamma nail is designed to permit the sliding of the lag screw, which enables it to apply compression on the fracture fragments that are accompanied with weight bearing. Appropriate compression on the fracture site enhances bone healing [13, 14]. The set screw locks the rotation and excessive telescoping of the lag screw. Nagura et al. reported that incorrect engagement of the set screw on the lag screw caused its loosening and complete deviation [15]. In the present case, the sliding of the lag screw was not restricted, and the lag screw migrated into the pelvis through the femoral head and medial wall of the acetabulum. The removed implant had no structural defects; however, the set screw was not inserted to the proper depth. The migration was found to be the result of the inappropriate insertion of the set screw to control the lag screw.

In previous reports, lag screws were removed from the femoral side in all cases. Tauber and Resch reported a case of sigmoid perforation after removing the migrated lag screw from the femoral side [16]. Thus, to avoid intestinal damage caused by the threaded part of the lag screw, we removed it from the pelvic side. Subsequently, no intestinal damage occurred during laparoscopic surgery. Similarly, it may be safer to remove the lag screw through laparoscopic surgery than through removal from the femoral side, because adhesions to the peritoneum or intestine can be directly observed.

In our case, we considered that the toggling caused by a combination of the greater trochanteric fragment and stovepipe canal, insufficient reduction, poor placement of the lag screw, and incorrect engagement of the set screw resulted in the medial pelvic migration of the lag screw.

Therefore, optimal fracture reduction and adequate positioning of the lag screw insertion are essential to avoid medial migration. Moreover, careful attention must be paid to subsequent steps such as set screw insertion to prevent complications. Surgeons should check radiographs regularly during patient follow-ups since some patients, such as those with schizophrenia, are less likely to feel the associated pain, as seen in the present case [17].

In conclusion, we experienced and reported a rare complication of postoperative lag screw migration into the pelvis with no trauma. The patient was treated with implant removal. This case indicates that optimal fracture reduction, adequate positioning of the lag screw, and careful attention to the set screw insertion are important to prevent complications.
